# AGO2 in T-prolymphocytic leukemia: its canonical and non-canonical deregulation and function

**DOI:** 10.18632/oncotarget.28378

**Published:** 2023-05-04

**Authors:** Till Braun, Hanna Klepzig, Marco Herling

**Keywords:** T-PLL, AGO2, microRNA

T-prolymphocytic leukemia (T-PLL) is a mature T-cell neoplasm with an aggressive and treatment-refractory course. In light of limited therapeutic options median overall survival times from diagnosis is hardly longer than 2 years [1]. There is currently no FDA- or EMA-approved drug for the treatment of T-PLL. Although 80–90% of patients experience a response to the most efficient single agent Alemtuzumab, relapses are common within the first 12–24 months following this first-line treatment.

One of the defining characteristics of T-PLL is the presence of the chromosomal aberrations *inv(14)* or *t(14;14)*, which lead to constitutive expression of the proto-oncogene *T-cell leukemia 1A* (*TCL1A*). This adapter molecule is centrally implicated in the enhanced T-cell receptor (TCR) signaling that is observed in the memory-type malignant T-cell [2]. Other recurrent genomic alterations that have been identified in T-PLL affect the genes *ataxia telangiectasia mutated* (*ATM*), *Janus kinase* (*JAK*), *signal transducer and activator of transcription* (*STAT*), and *MYC* [1].

In a recent study published by Braun et al. [3], we made significant advances in the understanding of the biology of T-PLL at the level of post-transcriptional gene regulation. For the first time, descriptive and mechanistic data implicated the involvement of molecules of the RNA interference (RNAi) machinery in T-PLL’s leukemogenesis and by that refined our current disease model by concepts beyond protein-coding genes. This work was preceded by piloting studies by our team [4] and others [5] on the differential profiles of microRNA (miR) expression in T-PLL, and already strongly implicated that pivotal pathways in T-PLL, such as TCR signaling, cell survival, and DNA-damage responses, are likely also shaped by such post-transcriptional deregulations.

**Figure 1:**
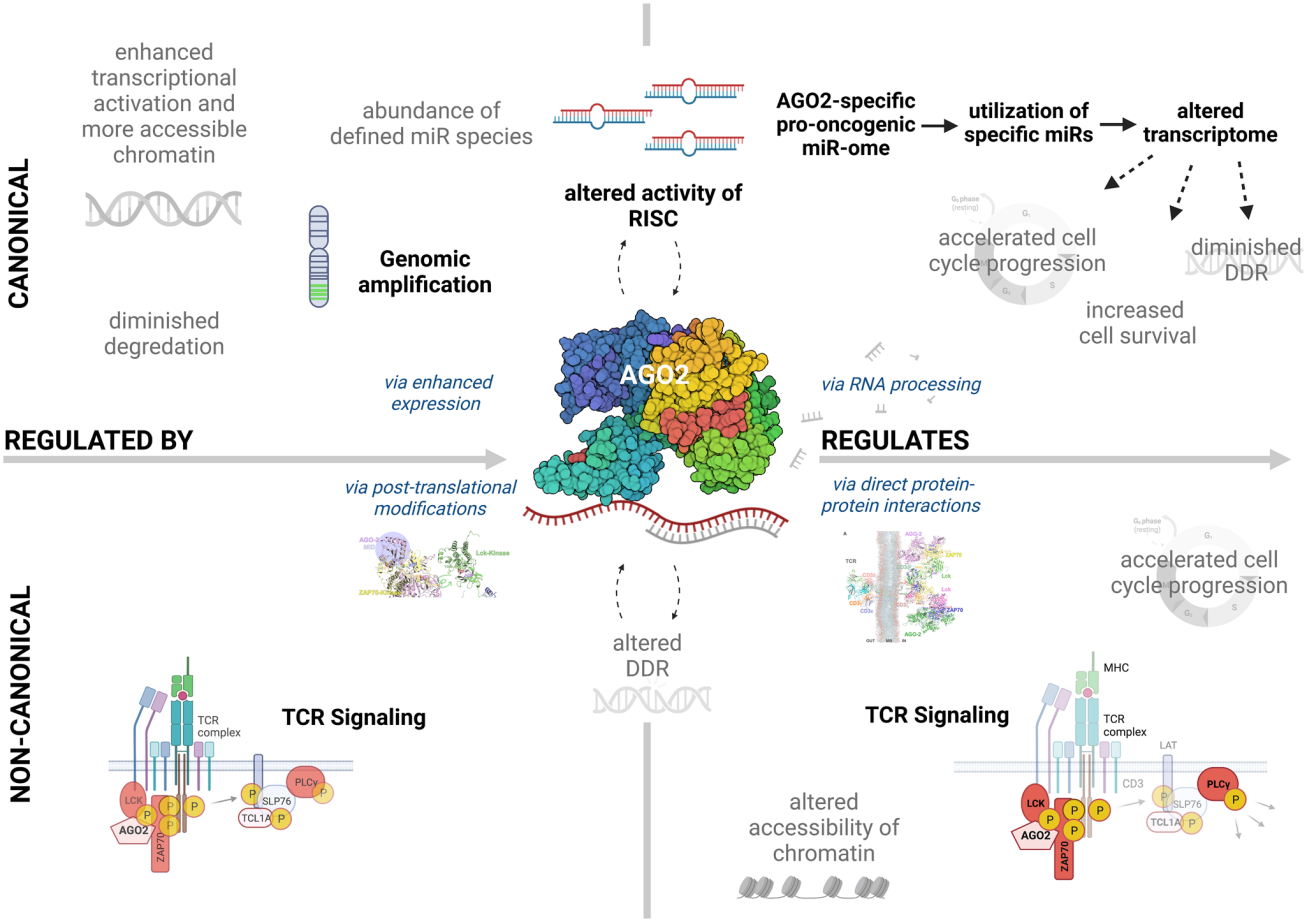
Scheme of mechanisms of AGO2 (de)regulation and its (novel) functions in T-PLL. Left: Canonical (top) as well as non-canonical (bottom) mechanisms of regulating the expression of AGO2 in T-PLL are presented: Besides genomic amplifications of chromosome 8q, observed in 29% of T-PLL cases, leading to overexpression of AGO2, there is upregulation of AGO2 protein by T-cell receptor (TCR) stimulation. In addition, three-dimensional structure models also suggest that AGO2 undergoes phosphorylation at Tyrosine (Y) 529 by the lymphocyte-specific protein tyrosine kinase (LCK) upon TCR activation, leading to enhanced TCR signaling capacity. DNA damage responses (DDR) are also implicated (via posttranslational modifications) as a non-conventional mechanism. Besides these three non-canonical regulatory mechanisms presented by Braun et al. [3], diminished degradation, increased transcriptional activation, epigenetic alterations, and the abundance of miR species are perceived as conventional regulatory factors of AGO2 expression (top). Right: Canonical (top) and non-canonical (bottom) functions of AGO2 in T-PLL: As a result of AGO2’s role in the RNA-induced silencing complex (RISC), we identified an AGO2-specific, pro-oncogenic miR-ome in T-PLL. By employing a cluster of specific microRNAs (miRs), AGO2 upregulation altered the transcriptome towards signatures of accelerated cell cycle progression, impaired DDR, and enhanced cell survival. Furthermore, we showed enhanced TCR signal strength upon AGO2 upregulation, particularly at the level of zeta-chain-associated protein kinase 70 (ZAP70), phospholipase C gamma 1 (PLCγ1), and linker for activation of T-cells (LAT) phospho-activation, as immediate, rather non-RNA mediated effects. We also gained first hints of an involvement of AGO2 in enhanced cell cycle progression, altered chromatin accessibility, and impaired DDRs, via direct protein-protein interactions as further non-canonical functions of AGO2 in T-PLL. Established mechanisms/functions shown in the publication by Braun et al. [3], are in bold, rather speculative mechanisms/functions are displayed in grey fond. AGO2 is presented in its crystal structure, displayed in a Van der Waal’s-based model. The figure was created with http://BioRender.com.

In Braun et al. [3], we then showed a significant upregulation of Argonaute 2 (AGO2), a central miR-regulating protein, in primary T-PLL cells. AGO2 is involved in the RNA-interference pathway and plays an essential role in miR-mediated gene silencing. Besides genomic amplifications of its locus at chromosome 8q, which we identified in 29% of patient samples, elevated AGO2 protein expression was proven in 73% of overall cases. Importantly, we unraveled TCR activation as a novel (non-canonical) way of AGO2 upregulation in T-PLL. In a model of leukemogenic AGO2 deregulation (Figure 1), we conceive as conventional regulatory factors of its protein expression (i) decreased degradation, (ii) increased transcriptional activation, (iii) epigenetic alterations, and (iv) abundance of defined miR species as well as non-conventional mechanisms (v) TCR signal input and (vi) an influence of DNA damage responses.

In line with AGO2’s function in the RNA-induced silencing complex (RISC), we demonstrated an enhanced miR-mediated mRNA degradation in T-PLL with high AGO2 protein expression through an integrated analysis of primary T-PLL transcriptomes and miR-omes in association with stratified AGO2 protein expression [3]. Additionally, we found that mRNAs and miRs associated with the HALLMARK pathways of survival signaling, cell cycle control, and DNA damage responses were significantly impacted by the expression of AGO2 (Figure 1). This finding builds on our previous work [4], in which we were able to characterize a pro-oncogenic T-PLL-specific miR-ome, and in which we have now outlined AGO2 as one of the determinants of such globally derailed miR profiles. Consequently, assessments of differences in miR loadings of AGO2 using AGO2/miR-immunoprecipitations should be among the tasks of future research in this area.

At the heart of a novel concept of AGO2 function, beyond its well-established role in global miR/mRNA network regulation, was our discovery that AGO2 also conveys rather immediate, non-RNA-mediated effects in T-PLL cells [3]. Using systems of genetically modulated AGO2, we observed a significant enhancement of TCR signal strength, specifically at the levels of kinase phospho-activation of the zeta-chain-associated protein kinase 70 (ZAP70), the phospholipase C gamma 1 (PLCγ1), and the linker for activation of T-cells (LAT) (Figure 1). Through comprehensive global mass-spectrometric analyses, we identified that the AGO2 protein in fact interacts with a unique set of partners in a TCR-stimulated context, including the central TCR kinases lymphocyte-specific protein tyrosine kinase (LCK) and ZAP70, forming membrane-associated protein complexes. Furthermore, our analysis of the three-dimensional structure of these complexes suggests AGO2 to undergo post-transcriptional modifications (PTMs) by LCK at its tyrosine (Y) 529 residue, which is predicted to be essential for ZAP70 binding. This PTM has previously been shown to be associated with a reduced small RNA binding, further pointing towards a non-canonical function of AGO2 when phosphorylated at Y529. However, this model is yet to be experimentally validated and we look forward to exploring this in future studies. Overall, these data shed new light on the understanding of T-PLL and also add to the increasing literature that highlights non-canonical functions of AGO2 in various malignancies [6].

In addition, our research has also laid the foundation for exciting new projects derived from the discovered concepts, i.e. around selective targeting of AGO2’s functions. For example, our studies uncovered that depletion of AGO2 in Jurkat cells leads to a significant increase in the expression of p27 in the TCR-unstimulated cells. As p27 is a well-established regulator of cell cycle and cell survival signaling, this increase in p27 expression is in line with our observation of reduced resistance to apoptosis in response to DNA damage induced by irradiation in these AGO2-depleted Jurkat cells. Moreover, we identified co-immunoprecipitation of AGO2 protein with PARP1, a key player in DNA repair, as well as with the cyclin-dependent kinase 1 (CDK1), a major determinant of proper cell cycle progression. This further points towards a robust link between AGO2 and DNA-damages responses, particularly at the level of cell cycle regulation (Figure 1). Future studies have to resolve the underlying mechanisms and contexts in more detail but have also to reveal whether these novel non-canonical (other than RISC-associated) functions of the AGO2 protein represent targetable vulnerabilities.

Taken together, there is a mutually influential relationship of AGO2 with the central growth and survival regulating TCR signaling in T-PLL. Especially by uncovering a previously unknown non-canonical function of the AGO2 protein that enhances TCR pathways in a RISC-independent manner, we added another factor to the concept of how the T-PLL cell or its precursor facilitates augmented TCR input, e.g., besides constitutive TCL1A or down-regulated CTLA4 [2]. This further highlights that TCR signaling intermediates, now including AGO2, represent potential targets to be explored in this aggressive and refractory disease [7].
